# Supplementary material for: High-resolution TADs reveal DNA sequences underlying genome organization in flies
Source: Nat Commun. 2018 Jan 15;9:189. doi: 10.1038/s41467-017-02525-w (PMC5768762; doi:10.1038/s41467-017-02525-w)
Supplement: Supplementary file 1 — Supplementary Information [file 41467_2017_2525_MOESM1_ESM.pdf]

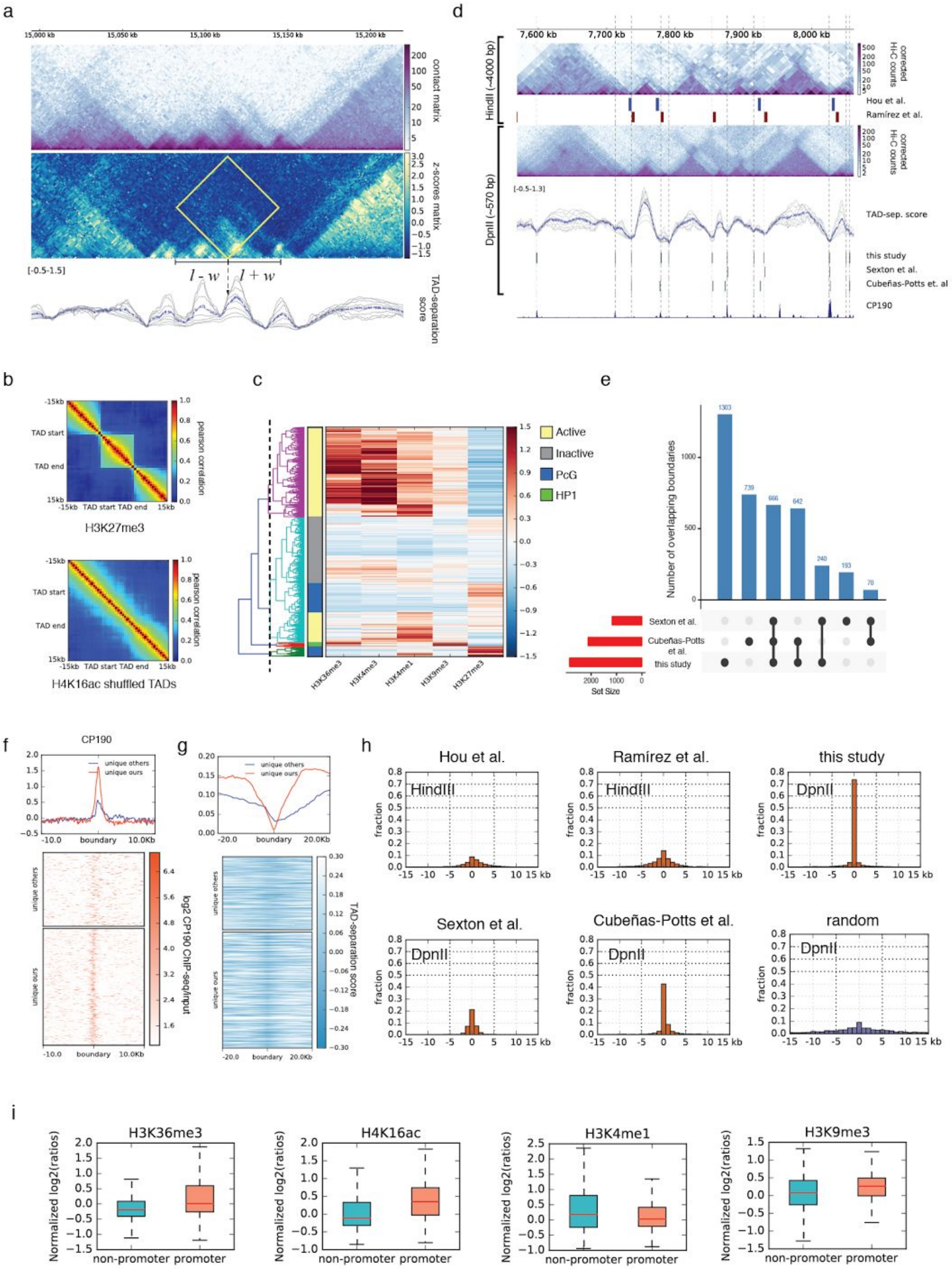
a
contact matrix
z-scores matrix
TAD-separation score
[-0.5-1.5]
l - w
l + w
d
HindIII (~4000 bp)
DpnII (~570 bp)
corrected Hi-C counts
Hou et al.
Ramírez et al.
TAD-sep. score
this study
Sexton et al.
Cubeñas-Potts et. al
CP190
b
H3K27me3
H4K16ac shuffled TADs
pearson correlation
TAD start
TAD end
c
Active
Inactive
PcG
HP1
H3K36me3
H3K4me3
H3K4me1
H3K9me3
H3K27me3
e
Number of overlapping boundaries
Sexton et al.
Cubeñas-Potts et al.
this study
Set Size
f
CP190
unique others
unique ours
boundary
log2 CP190 ChIP-seq/input
g
TAD-separation score
h
Hou et al.
Ramírez et al.
this study
Sexton et al.
Cubeñas-Potts et al.
random
HindIII
DpnII
fraction
i
H3K36me3
H4K16ac
H3K4me1
H3K9me3
Normalized log2(ratios)
non-promoter
promoter

Supplementary Figure 1. **Detection of TAD boundaries and assessment of their quality**. **a.** Method to identify boundaries (see 'methods' for details). Top, Hi-C contact matrix counts. Middle, z-score transformed matrix. Last: TAD-separation score at different window lengths (gray lines) and mean score (blue line). The TAD-separation score at bin (l) corresponds to the mean values of the z-scores inside the 'diamond', which correspond to the contacts between a region of width w on left and on right. **b.** Similar to Fig. 1c, Pearson correlations of histone marks within and outside TADs (+15kb flanking regions) and for a random placement of boundaries. **c.** Hierarchical clustering of TADs based on modENCODE histone marks for Kc167 cells. Manual annotations for active, inactive, PcG and HP1 are based on the clustering results (see methods). **d.** Example genomic location comparing published boundaries with the ones generated in this study. Top, HindIII based Hi-C contact matrix counts for S2 cell line [1] and boundaries reported by Hou et al.[2] (Kc167) and Ramirez et al.[1] (S2). Bottom, DpnII based Hi-C contact matrix and the boundaries from this study, Sexton et al.[3] and Cubeñas-Potts et al. [4]. Below the Hi-C heatmap is the TAD-separation score as in Fig. 1a. The last track shows CP190 ChIP-seq signal [5].The vertical lines correspond to the boundaries in this study. **e.** Overlap of boundaries based on Hi-C DpnII experiments. The bars show the overlap between the indicated sets below (black dots). Two boundaries were considered overlapping if they were within 2000 bp from each other. The intersections were plotted using UpSetR [1]. **f.** Comparison of unique boundaries in our study with the unique boundaries in previous studies [2,3] with respect to CP190. Our unique boundaries overlap more frequently with CP190. **g.** Similar to f, but comparing TAD separation score. **h.** Histograms of the distance of our boundaries and other published boundary sets to CP190 peaks. The 'random' dataset contains our boundary set randomly shuffled (see methods). **i.** As in Fig. 1h, modENCODE histone marks at non-promoter and promoter boundaries. In all cases the promoter boundaries are associates significantly to the active marks (H3K36me3 and H4K16ac, p-value <=3.106261e-17 Wilcoxon rank-sum test).

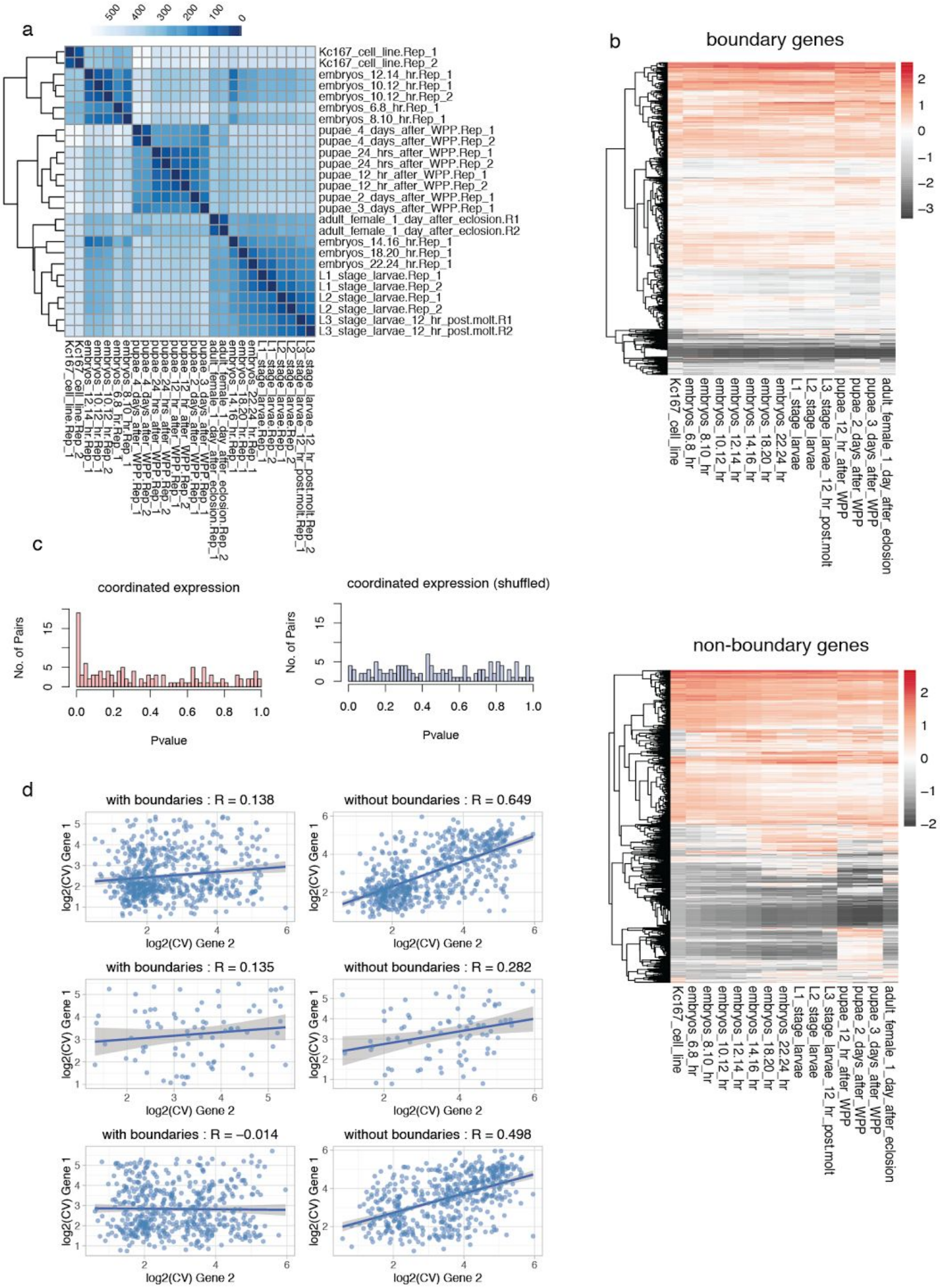
a
500
400
300
200
100
0
Kc167_cell_line.Rep_1
Kc167_cell_line.Rep_2
embryos_12.14_hr.Rep_1
embryos_10.12_hr.Rep_1
embryos_10.12_hr.Rep_2
embryos_6.8_hr.Rep_1
embryos_8.10_hr.Rep_1
pupae_4_days_after_WPP.Rep_1
pupae_4_days_after_WPP.Rep_2
pupae_24_hrs_after_WPP.Rep_1
pupae_24_hrs_after_WPP.Rep_2
pupae_12_hr_after_WPP.Rep_1
pupae_12_hr_after_WPP.Rep_2
pupae_2_days_after_WPP.Rep_1
pupae_3_days_after_WPP.Rep_1
adult_female_1_day_after_eclosion.R1
adult_female_1_day_after_eclosion.R2
embryos_14.16_hr.Rep_1
embryos_18.20_hr.Rep_1
embryos_22.24_hr.Rep_1
L1_stage_larvae.Rep_1
L1_stage_larvae.Rep_2
L2_stage_larvae.Rep_1
L2_stage_larvae.Rep_2
L3_stage_larvae_12_hr_post.molt.R1
L3_stage_larvae_12_hr_post.molt.R2
b
boundary genes
2
1
0
−1
−2
−3
Kc167_cell_line
embryos_6.8_hr
embryos_8.10_hr
embryos_10.12_hr
embryos_12.14_hr
embryos_14.16_hr
embryos_18.20_hr
embryos_22.24_hr
L1_stage_larvae
L2_stage_larvae
L3_stage_larvae_12_hr_post.molt
pupae_12_hr_after_WPP
pupae_2_days_after_WPP
pupae_3_days_after_WPP
adult_female_1_day_after_eclosion
non-boundary genes
2
1
0
−1
−2
c
coordinated expression
coordinated expression (shuffled)
No. of Pairs
Pvalue
d
with boundaries : R = 0.138
without boundaries : R = 0.649
with boundaries : R = 0.135
without boundaries : R = 0.282
with boundaries : R = −0.014
without boundaries : R = 0.498
log2(CV) Gene 1
log2(CV) Gene 2

Supplementary Figure 2. Gene expression is coordinated inside TADs. a. Euclidean distances between RNA-Seq samples from modENCODE used in this study. Replicates were later merged (mean) for the analysis. **b.** Clustering of genes by expression in Kc167 cells and at different developmental stages. Genes lying on either side of TAD boundaries tend to show consistent expression (top), while genes within TADs show variable expression (bottom) during development (color bar : row-wise z-score). Genes without boundaries were sampled randomly to the same number as genes with boundaries. **c.** P-values from ANOVA between genes within pairs of adjacent TADs (see methods). Expression within TADs is more coordinated (left) compared to genes randomly assigned to TADs (right). **d.** Same as Fig. 2d, here the adjacent gene-pairs are separated by their relative orientation: divergent (top), convergent (middle), and tandem (below) pairs. Gene-pairs without boundaries were sampled randomly to the same number as genes with boundaries. Line shows the linear model fit (shaded region: std. error).

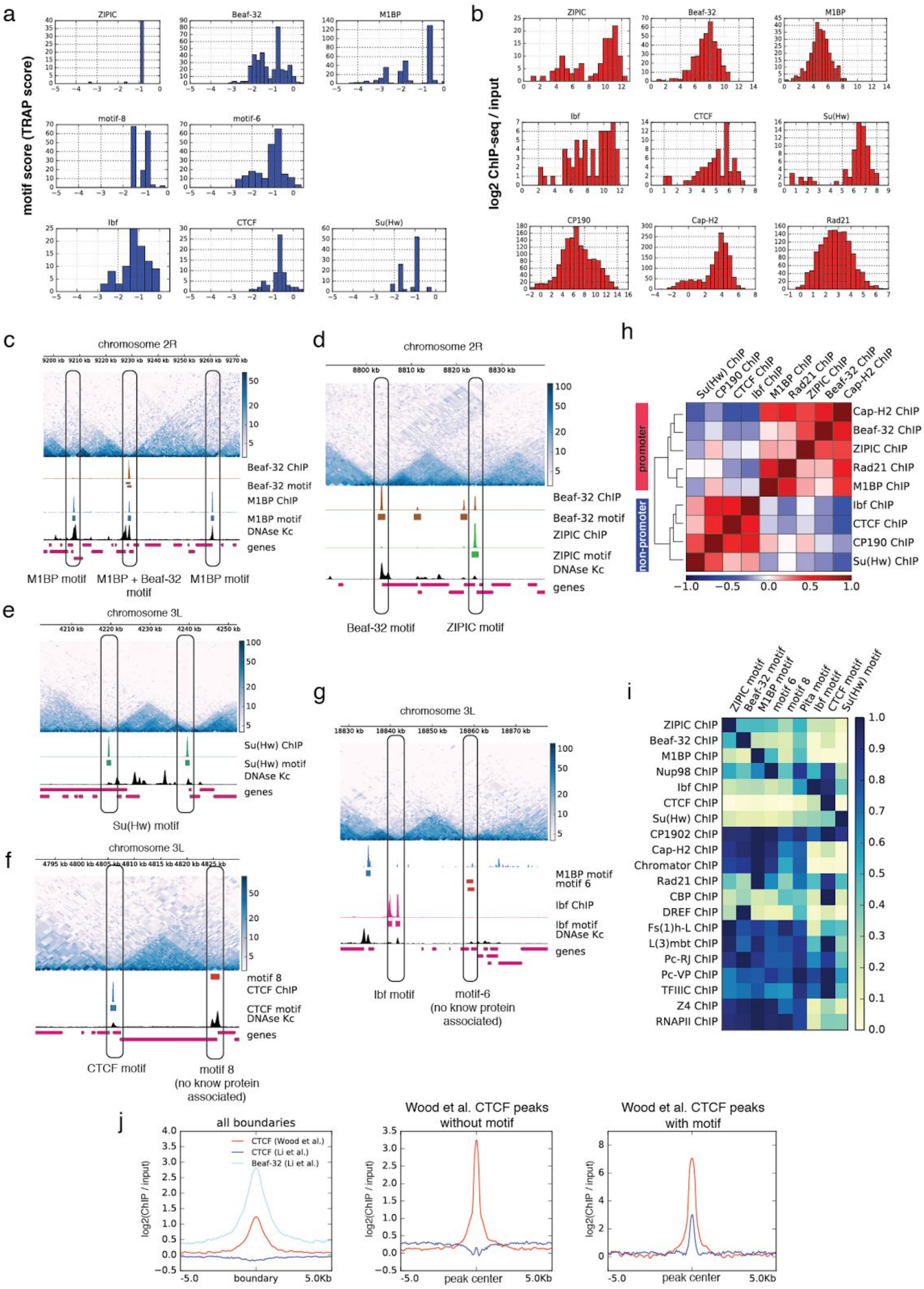
a
motif score (TRAP score)
ZIPIC
Beaf-32
M1BP
motif-8
motif-6
Ibf
CTCF
Su(Hw)
b
log2 ChIP-seq / input
ZIPIC
Beaf-32
M1BP
Ibf
CTCF
Su(Hw)
CP190
Cap-H2
Rad21
c
chromosome 2R
Beaf-32 ChIP
Beaf-32 motif
M1BP ChIP
M1BP motif
DNAse Kc
genes
M1BP motif
M1BP + Beaf-32 motif
M1BP motif
d
chromosome 2R
8800 kb
8810 kb
8820 kb
8830 kb
Beaf-32 ChIP
Beaf-32 motif
ZIPIC ChIP
ZIPIC motif
DNAse Kc
genes
Beaf-32 motif
ZIPIC motif
h
promoter
non-promoter
Su(Hw) ChIP
CP190 ChIP
CTCF ChIP
Ibf ChIP
M1BP ChIP
Rad21 ChIP
ZIPIC ChIP
Beaf-32 ChIP
Cap-H2 ChIP
Cap-H2 ChIP
Beaf-32 ChIP
ZIPIC ChIP
Rad21 ChIP
M1BP ChIP
Ibf ChIP
CTCF ChIP
CP190 ChIP
Su(Hw) ChIP
e
chromosome 3L
4210 kb
4220 kb
4230 kb
4240 kb
4250 kb
Su(Hw) ChIP
Su(Hw) motif
DNAse Kc
genes
Su(Hw) motif
g
chromosome 3L
18830 kb
18840 kb
18850 kb
18860 kb
18870 kb
M1BP motif
motif 6
Ibf ChIP
Ibf motif
DNAse Kc
genes
Ibf motif
motif-6 (no know protein associated)
f
chromosome 3L
motif 8
CTCF ChIP
CTCF motif
DNAse Kc
genes
CTCF motif
motif 8 (no know protein associated)
i
ZIPIC motif
Beaf-32 motif
M1BP motif
motif 6
motif 8
Pita motif
Ibf motif
CTCF motif
Su(Hw) motif
ZIPIC ChIP
Beaf-32 ChIP
M1BP ChIP
Nup98 ChIP
Ibf ChIP
CTCF ChIP
Su(Hw) ChIP
CP1902 ChIP
Cap-H2 ChIP
Chromator ChIP
Rad21 ChIP
CBP ChIP
DREF ChIP
Fs(1)h-L ChIP
L(3)mbt ChIP
Pc-RJ ChIP
Pc-VP ChIP
TFIIIC ChIP
Z4 ChIP
RNAPII ChIP
j
all boundaries
CTCF (Wood et al.)
CTCF (Li et al.)
Beaf-32 (Li et al.)
log2(ChIP / input)
boundary
Wood et al. CTCF peaks without motif
peak center
Wood et al. CTCF peaks with motif
peak center

Supplementary Figure 3. Boundary motifs and their relationship to the ChIP-Seq profiles. **a.** TRAP scores for each of the TAD boundary motifs within their cluster. **b**. ChIP-seq log2 ratio (ChIP/input) for the insulator proteins within their cluster. In the case of CP190, Cap-H2 and Rad21 the histogram contains the values over all boundaries. **c-g.** Examples of distinct insulators at boundaries**.** As in Fig. 1a, the top track shows Hi-C corrected counts. **h.** Heatmap showing Pearson correlations of ChIP-seq log2 ratios (IP / input) measured at TAD boundaries. The *complete* linkage method (also known as furthest neighbour clustering) was used for the hierarchical clustering. **i.** As in Fig. 3d. Each cell in the matrix contains the mean fold change of all respective ChIP-seq peaks having the motif. For each row, the maximum fold change was scaled to 1. **j.** Comparison of CTCF ChIP-seq experiments from Wood et. al.[4] and Li et. al.[5]. The first panel contains the mean values over all boundaries, the middle panel contains mean values for all CTCF peaks from Wood et. al.[4] that do not have the CTCF motif, and the last panel contains CTCF peaks from Wood et. al.[4] that have the CTCF motif. The CTCF ChIP-seq from Li et. al.[5] only shows enrichment when the CTCF motif is present while the CTCF ChIP-seq from Wood et al.[6] has unspecific bindings.

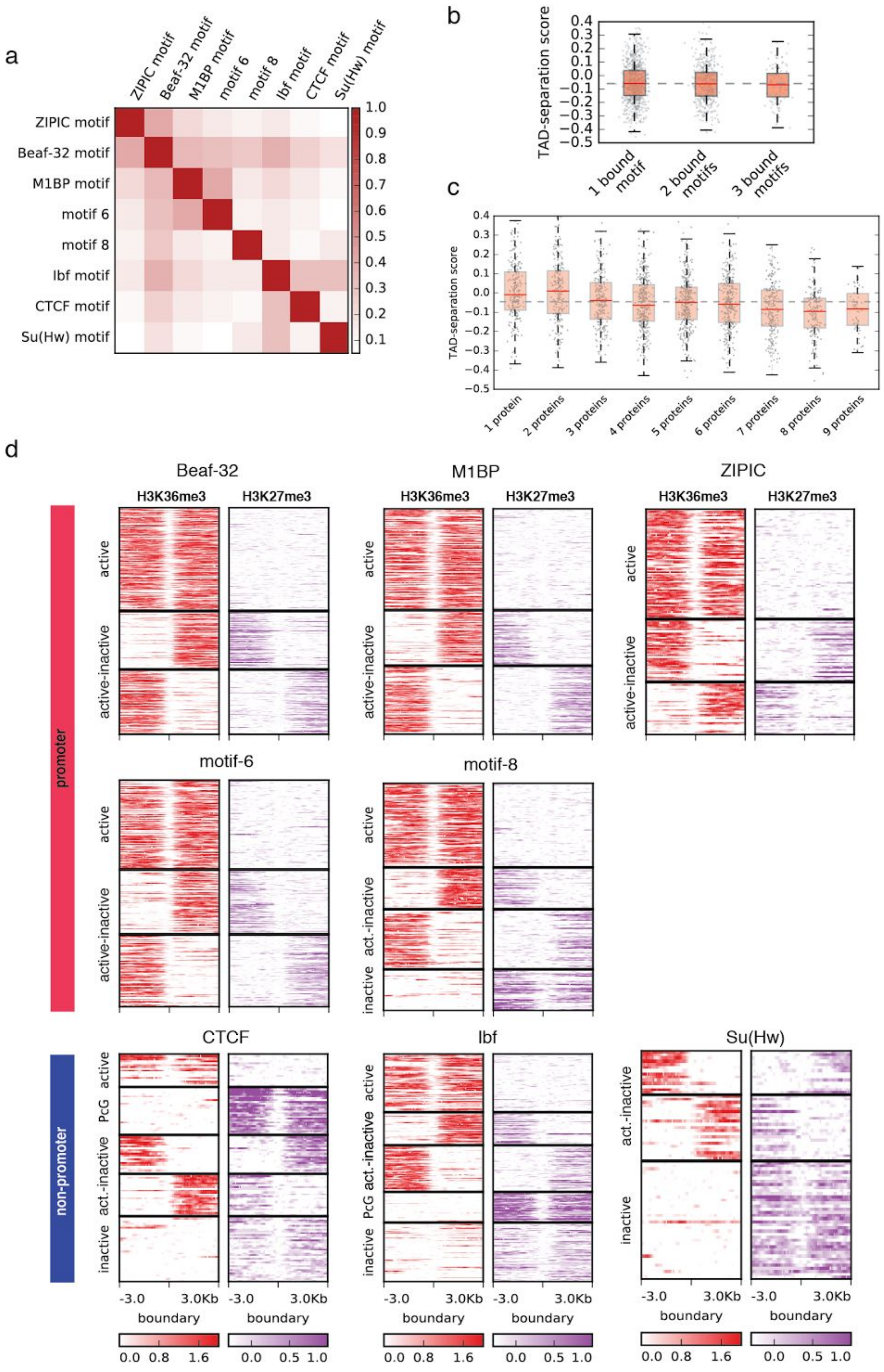
a
ZIPIC motif
Beaf-32 motif
M1BP motif
motif 6
motif 8
Ibf motif
CTCF motif
Su(Hw) motif
b
TAD-separation score
1 bound motif
2 bound motifs
3 bound motifs
c
TAD-separation score
1 protein
2 proteins
3 proteins
4 proteins
5 proteins
6 proteins
7 proteins
8 proteins
9 proteins
d
Beaf-32
M1BP
ZIPIC
H3K36me3
H3K27me3
motif-6
motif-8
CTCF
Ibf
Su(Hw)
promoter
non-promoter
active
active-inactive
act.-inactive
inactive
PcG
-3.0
3.0Kb
boundary

Supplementary Figure 4. **Effect of motif combinations on boundary strength and chromatin marks. a**. Heatmap of the overlap coefficient between boundary motifs. For each pair of motifs, the overlap coefficient is defined as Overlap-coefficient$(A, B) = A \cap B / \min(|A|, |B|)$ where A, B are the sets of all boundaries containing either motif A or B. **b**. TAD separation at boundaries containing one, two or three insulator motifs. Virtually no difference in boundary strength is observed with the number of insulator motifs present at boundaries. **c.** TAD separation score at boundaries bound by 1 up to 10 proteins known to be associated with boundaries (discarding information about motif enrichment). If we do not consider motif information, some variation can be seen in boundary strength associated to the number of bound proteins, especially between 2 to 3 boundary proteins and between 6 and 7 proteins (*p*-value <= 0.01 Wilcoxon rank sum test). **d.** Normalized log2 ChIP/input at transitions from different histone marks at boundaries for the active chromatin H3K36me3 and the repressive chromatin mark H3K27me3 on a 6 kb region centered at the boundaries. We performed *k*-means clustering using deepTools [6]. For CTCF and Ibf five clusters were used to distinguish boundaries between Polycomb group TADs. The low histone mark values at the boundaries (white color running at the center of the heatmaps) are indicative of nucleosome free regions. The polycomb group repressed chromatin (PcG) is characterized by higher intensities of H3K27me3 compared to the inactive chromatin.

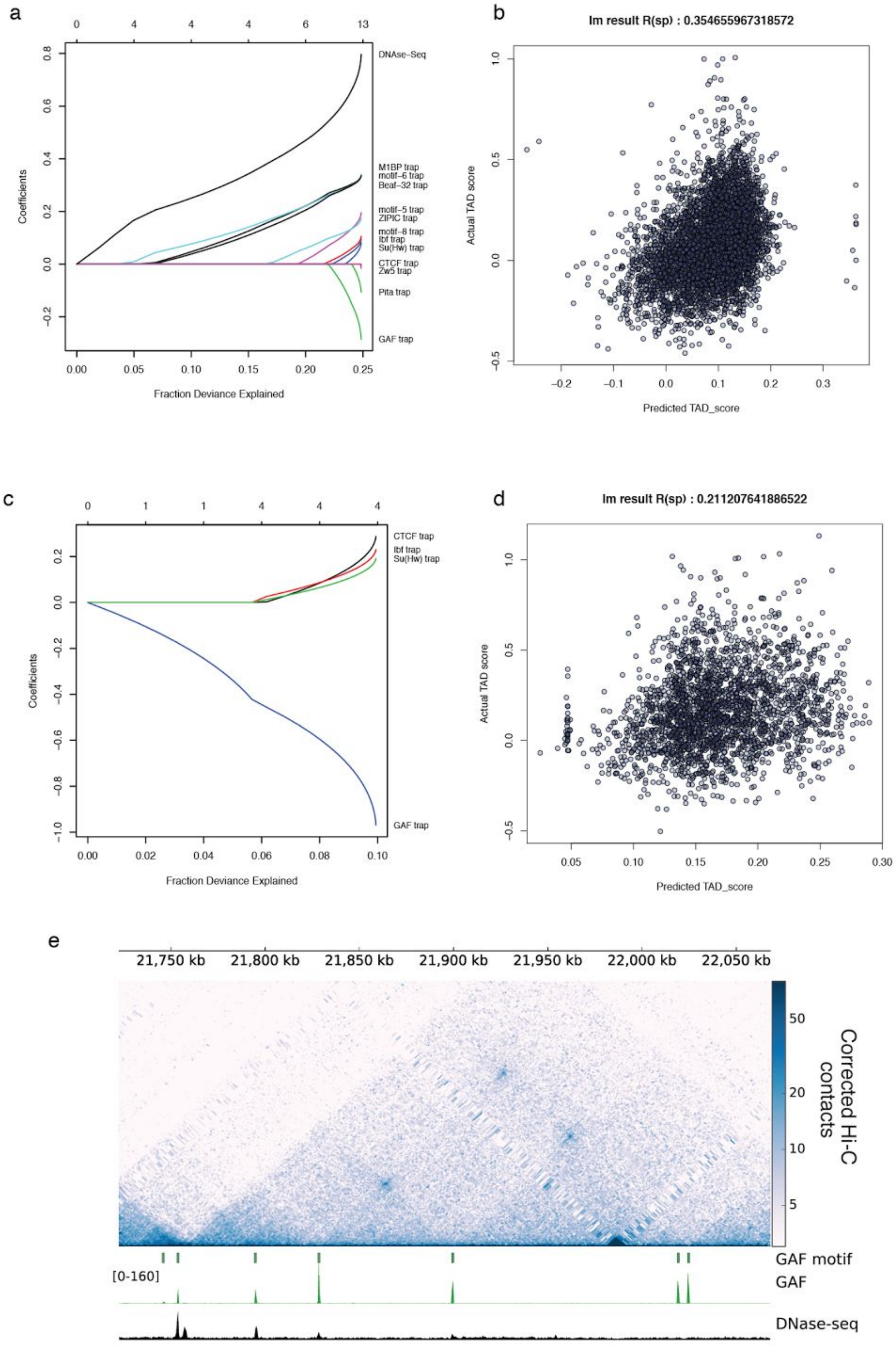
a
b
lm result R(sp) : 0.354655967318572
c
d
lm result R(sp) : 0.211207641886522
e
Coefficients
Fraction Deviance Explained
DNAse-Seq
M1BP trap
motif-6 trap
Beaf-32 trap
motif-5 trap
ZIPIC trap
motif-8 trap
Ibf trap
Su(Hw) trap
CTCF trap
Zw5 trap
Pita trap
GAF trap
Actual TAD score
Predicted TAD_score
21,750 kb
21,800 kb
21,850 kb
21,900 kb
21,950 kb
22,000 kb
22,050 kb
Corrected Hi-C contacts
GAF motif
[0-160]
GAF
DNase-seq

Supplementary Figure 5. Results from lasso and linear model predictions. a. Lasso penalized coefficients for promoters. Open chromatin (DNAse-seq), followed by Beaf-32, M1BP and motif-6 are the three top predictors to classify promoters as TAD boundaries, while GAF and Pita are negatively associated. **b**. At promoters: TAD score predictions using linear model on an independent test dataset. The predicted scores correlate with the actual TAD-separation scores on promoters. **c**. Lasso penalized coefficients for non-promoter open sites. TRAP score signal for CTCF, Ibf1 and Su(Hw) are positively correlated with boundaries, while GAF shows negative correlation. **d**. At non-promoter open sites : predicted TAD scores from linear model on an independent test dataset. **e**. GAF motif, which is negatively associated with promoter and non-promoter boundaries, can be found alone along with the protein at loop domains.

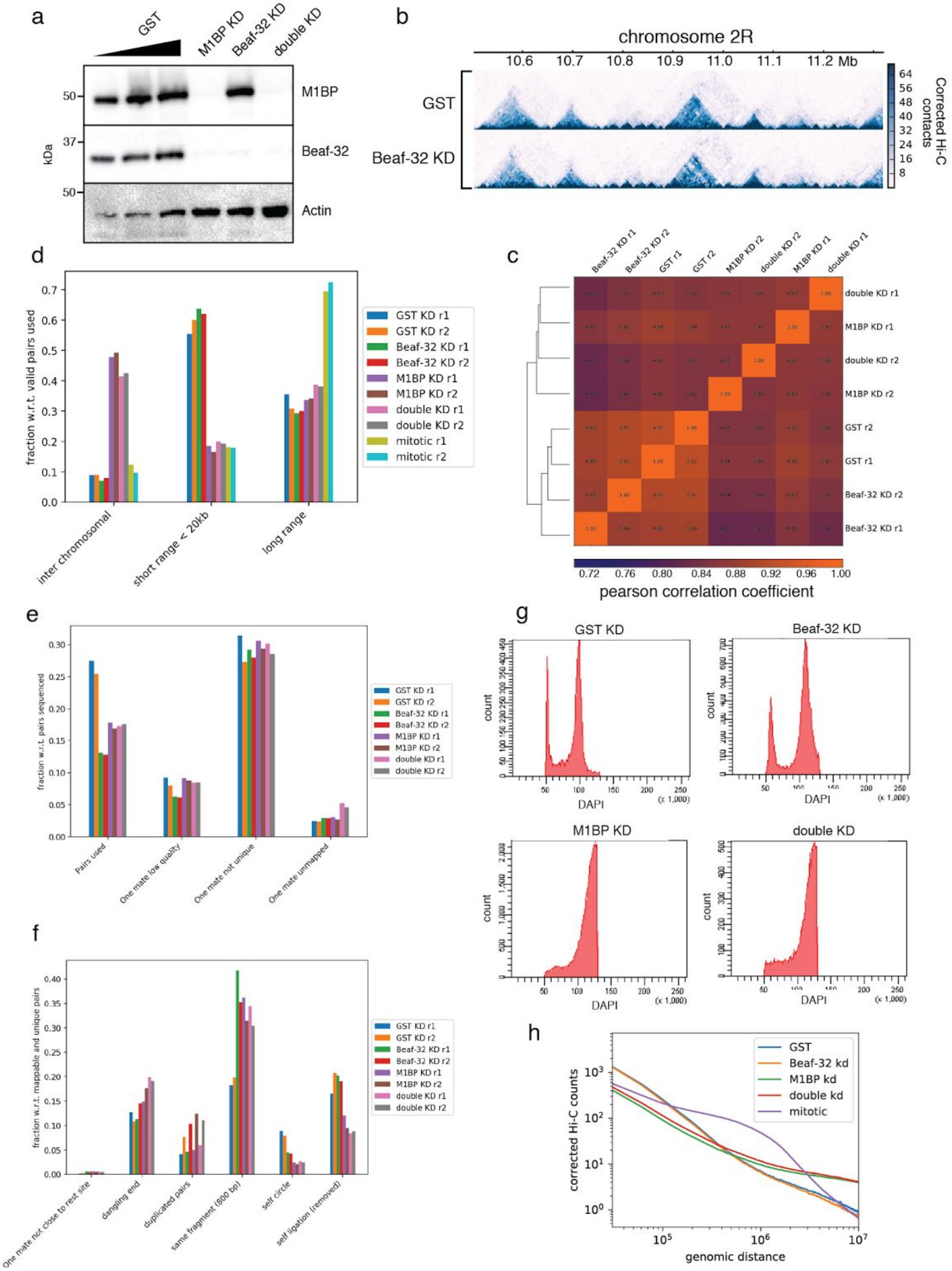
a
GST
M1BP KD
Beaf-32 KD
double KD
50
37
50
kDa
M1BP
Beaf-32
Actin
b
chromosome 2R
10.6
10.7
10.8
10.9
11.0
11.1
11.2 Mb
GST
Beaf-32 KD
Corrected Hi-C contacts
64
56
48
40
32
24
16
8
c
Beaf-32 KD r1
Beaf-32 KD r2
GST r1
GST r2
M1BP KD r2
double KD r2
M1BP KD r1
double KD r1
pearson correlation coefficient
0.72
0.76
0.80
0.84
0.88
0.92
0.96
1.00
d
fraction w.r.t. valid pairs used
inter chromosomal
short range < 20kb
long range
GST KD r1
GST KD r2
Beaf-32 KD r1
Beaf-32 KD r2
M1BP KD r1
M1BP KD r2
double KD r1
double KD r2
mitotic r1
mitotic r2
e
fraction w.r.t. pairs sequenced
Pairs used
One mate low quality
One mate not unique
One mate unmapped
f
fraction w.r.t. mappable and unique pairs
One mate not close to rest site
dangling end
duplicated pairs
same fragment (800 bp)
self circle
self ligation (removed)
g
GST KD
Beaf-32 KD
M1BP KD
double KD
count
DAPI
(x 1,000)
h
corrected Hi-C counts
genomic distance
GST
Beaf-32 kd
M1BP kd
double kd
mitotic

Supplementary Figure 6. Beaf-32 and M1BP knockdowns Hi-C. a. Western blots showing protein levels of M1BP and Beaf-32 (compared to GST control) after RNAi treatment in S2 cells. **b**. Example region showing contacts for all GST and Beaf-32 KD. All matrices were normalized to match the total number of contacts of the smaller matrix. The bin size used was 3kb. **c**. Pearson correlation of corrected Hi-C matrices. The correlation was done between Hi-C bins within 50 kbp. **d.** Fraction of Hi-C pairs classified as inter-chromosomal by *cis* distancer. Mitotic data values were added for comparison from Hug et al.[7] The total number of reads sequenced are listed in Supplementary Table 5. **e.** Fraction of valid Hi-C pairs used compared to low quality, unmapped and non-unique. **f**. Fraction of Hi-C pairs that are filtered by various reasons. These plots is part of the QC module of HiCExplorer. **g**. FACS analysis using DAPI for GST, Beaf-32, M1BP and M1BP + Beaf-32 knockdowns. M1BP knockdown affects cell growth and causes an arrest in cell-cycle not seen in GST or Beaf-32 KD. **h**. Genomic distance vs. Hi-C counts. Larger matrices were scaled down to match the sum of the smallest matrix. Only replicate 1 of all experiments was used. The 'mitotic' data is based on the Hi-C data on mitotic fly cells from Hug et. al. [7].

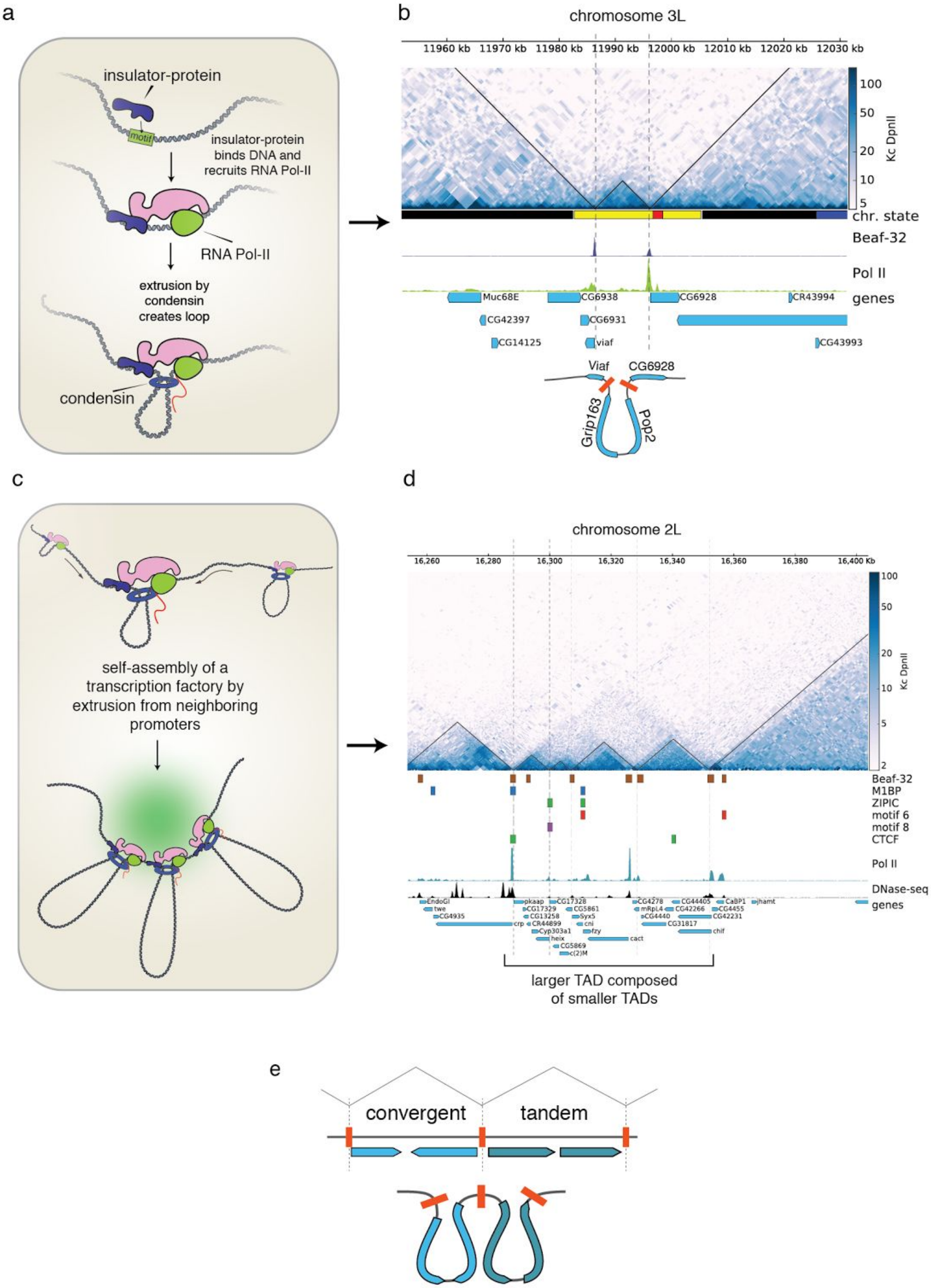
a
insulator-protein
motif
insulator-protein binds DNA and recruits RNA Pol-II
RNA Pol-II
extrusion by condensin creates loop
condensin
b
chromosome 3L
11960 kb
11970 kb
11980 kb
11990 kb
12000 kb
12010 kb
12020 kb
12030 kb
Kc DpnII
100
50
20
10
5
chr. state
Beaf-32
Pol II
genes
Muc68E
CG6938
CG6928
CR43994
CG42397
CG6931
CG14125
viaf
CG43993
Viaf
CG6928
Grip163
Pop2
c
self-assembly of a transcription factory by extrusion from neighboring promoters
d
chromosome 2L
16,260
16,280
16,300
16,320
16,340
16,360
16,380
16,400 Kb
Kc DpnII
100
50
20
10
5
2
Beaf-32
M1BP
ZIPIC
motif 6
motif 8
CTCF
Pol II
DNase-seq
genes
larger TAD composed of smaller TADs
e
convergent
tandem

Supplementary Figure 7. Model for the formation of TADs. a. Top: Binding of an insulator protein to its cognate motif at at core promoter facilitates RNA Pol-II initiation which in turn recruits condensin complex which initiates extrusion [8]. **b.** As shown by simulations [9,10] TADs in Hi-C contact maps are consistent with loop extrusion. Here we show a small active region between two large inactive TADs. The active region contains two genes and is flanked by the Beaf-32 insulator. The expected loop from this region is shown below. **c.** Neighboring promoters, each extruding a loop, end up meeting each other to form a rosette reminiscent of proposed transcription factories [11,12]. Such clusters, are also observed in a recent single-nucleus HiC study [13]. **d.** The TADs demarcated by the insulators motif and RNA Pol-II form a larger TAD. This implies that the smaller TADs remain in closer contact to each other compared to the surrounding inactive chromatin. This hierarchical TAD structure resembles the proposed structure of transcription factories. **e.** Insulators at divergent gene promoters serve as good anchors, by recruiting two Pol-II/Condensin machines in both directions. Red bars represent insulator motifs at promoters and dotted lines show the location of boundaries. The expected loops from this region are shown below.

Supplementary Table 1. Enriched motifs found at ChIP-seq peaks.

MOTIF Protein	Beaf-32	M1BP	Pita	CTCF	Ibf1/2	ZIPIC	Zw5	Su(Hw)	GAF	CP190	Mod (mdg4)	Cap-H2	Chro-mator	Rad21
Beaf-32 motif	285(600) 3.3e-230	95(600) 4.2e-18	85(600) 1.2e-014			119(600) 5.2e-72				51(300) 2.8e-5		219(600) 3.0e-187	102(300) 5.5e-52	28(300) 9.9e-8
M1BP motif	30(600) 9.5e-7	519(600) 6.0e-1012	73(600) 8.4e-054			47(600) 2.3e-6	28(300) 2.1e-10			88(300) 3.3e-42		102(600) 3.4e-98	88(300) 1.2e-074	84(300) 4.2e-97
Pita motif			114(600) 1.1e-093		56 (300) 3.8e-21									
CTCF motif	24(600) 1.1e+3		74(600) 1.1e-53	272(600) 5.0e-562	75(300) 9.3e-92	73(600) 1.2e-22				32(300) 1.0e-20	42(300) 9.4e-44			
Ibf1/2 motif				3.5e-008 (dreme)	195(300) 2.6e-49					2.5e-9 (dreme)				
ZIPIC motif	168(600) 7.6e-55	19(600) 1.4	108(600) 3.3e-108			356(600) 6.1e-256				28(300) 3.6e-4		120(600) 1.2e-61		
Zw5 motif							87(300) 3.1e-98							
Su(Hw) motif								175(300) 3.8e-208		66(300) 7.1e-60	27(300) 9.4e-13		24(300) 5.4e-9	
GAF motif									67(300) 1.8e-152		27(300) 9.4e-13			
Ohler motif 5 motif	40(600) 3.5e+6	27(600) 5.5e-1	48(600) 1.8e-21			71(600) 2.7e-23	27(300) 8.9e-12			26(300) 5.3e-6				
Ohler motif 6 motif		138(600) 9.6e-94				40(600) 3.0e-11				67(300) 4.0e-10		67(600) 4.8e-14		
Ohler motif 8 motif	30(600) 9.5e-7	12(600) 6.1e+8			54(300) 1.0e-7	30(600) 4.7e+3				49(300) 1.6e-2	42(300) 3.4e-9	18(600) 2.4e-12		98(300) 3.0e-7
A-rich repeat									128(300) 6.4e-15				118(300) 2.8e-105	
(CA)n repeat						40(600) 1.0e-11		43(300) 1.7e-42	77(300) 2.5e-276					35(300) 2.0e-22

The table shows number of sites with motifs (total number of sites) and MEME[14] E-value. In some cases the DREME[15] E-value is shown.

Supplementary Table 2. Comparison of motif enrichment using different sets of boundaries.

	DE-NOVO						KNOWN			
**MOTIF**	**AME all**	**TRAP all**	**AME Non promoters**	**TRAP Non promoters**	**Meme Non promoters**	**Dreme Non promoters**	**AME all**	**TRAP all**	**AME Non promoters**	**TRAP Non promoters**
**Sexton et al.**										
**M1BP**	6.36E-29	1.49E-22					9.11E-36	2.67E-27		
**Beaf-32**	1.72E-21	1.87E-23					6.02E-22	4.07E-22		
**Motif-6**	1.39E-11	5.73E-17					6.50E-16	9.86E-18		
**ZIPIC**	NA	NA					6.71E-15	1.87E-10		
**Motif-8**	NA	NA					3.34E-04	2.9E-5		
**CTCF**	NA	NA	NA		4.00E+10		NA	0.96	5.04E-09	1.4E-4
**Su(Hw)**	NA	NA	2.91E-01	0.0313	6.20E+14		NA	0.082	2.49E-02	0.0381
**Ibf1/2**	NA	NA	NA				2.22E-04	1.3E-3	NA	6.6E-4
**Cubeñas-Potts et al.**										
**M1BP**	1.17E-50	5.90E-34					8.65E-53	6.38E-40		
**Beaf-32**	1.25E-52	1.04E-50					1.45E-46	1.16E-48		
**Motif-6**	9.64E-38	1.52E-45					2.23E-38	3.91E-43		
**ZIPIC**	NA	NA					8.10E-27	1.07E-19		
**Motif-8**	NA	NA					1.47E-10	9.12E-11		
**CTCF**	NA	NA	8.10E-11	0.00129	9.17E-06		NA	0.117	7.53E-06	0.0633
**Su(Hw)**	NA	NA	NA	6.79E-06			3.69E-02	3.5E-4	1.52E-06	0.03481
**Ibf1/2**	NA	NA	NA				1.08E-11	2.75E-09	9.03E-02	6.53E-05
**This study**										
**M1BP**	4.24E-72	9.96E-72					7.84E-85	5.20E-87		
**Beaf-32**	2.10E-64	1.27E-74					1.63E-64	3.27E-70		
**Motif-6**	4.83E-45	5.26E-64					1.47E-48	8.65E-66		
**ZIPIC**	NA	NA					5.09E-37	1.07E-30		
**Motif-8**	1.58E-04	0.721					8.86E-08	7.27E-16		
**CTCF**	NA	NA	1.83E-04	0.0511	1.10E-02	1.10E-06	NA	0.095184	7.53E-06	0.0636
**Su(Hw)**	NA	0.0987	2.09E-03	3.89E-23	NA	2.10E-04	3.78E-03	4.30E-07	1.52E-06	3.87E-10
**Ibf**	NA	NA	NA	0.00137	NA		3.16E-07	6.22E-10	9.03E-02	2.03E-05

Supplementary Table 3. Hi-C data sources

Source	Restriction enzyme	No. of usable reads	GEO accession	Reference
Whole embryos	DpnII	133.483.965	GSE34453	[2]
Kc167	DpnII	135.274.348	GSE63515	[16]
Kc167	DpnII	110.807.526	GSE80701	[3]
Kc167	HindIII	71.278.991	GSE38468	[17]
S2	HindIII	680.121.887	GSE58821	[18]
Clone-8	HindIII	131.426.003	GSE58821	[18]
third instar larvae salivary glands	HindIII	9.404.794	GSE72512	[19]

Supplementary Table 4. ChIP-seq data sources.

Source	GEO accession	Reference
Kc167 Beaf-32	GSM762845	[4]
Kc167 CP190	GSM762836	[4]
Kc167 CTCF	GSM1535983	[16]
Kc167 Su(Hw)	GSM762839	[4]
Kc167 Cap-H2	GSM1318356	[20]
Kc167 Chromator	GSM1318357	[20]
Kc167 Rad21	GSM1318352	[20]
Kc167 Pita	GSM2133768	[3]
Kc167 ZIPIC	GSM2133769	[3]
Kc167 GAF	GSM2133762	[3]
Kc167 Ibf 1	GSM2133766	[3]
Kc167 Ibf 2	GSM2133767	[3]
Embryo Zw5	GSM2042227	[21]
S2 M1BP	GSM1208162	[22]
Kc167 RNA Pol-II	GSM1536014	[16]
S2 DNase-seq	GSM1000406	[23]

Supplementary Table 5. Number of sequencing reads for Hi-C control and knockdowns.

	GST rep. A	GST rep. B	Beaf-32 KD rep. A	Beaf-32 KD rep. B	M1BP KD rep. A	M1BP KD rep. B	double KD rep. A	double KD rep. B
**Pairs considered**	107.8M	118.5M	102.4M	151.0M	298.1M	213.8M	217.3M	180.5M
**Pairs used**	32.1M	32.5M	14,5M	22.0M	58.7M	40.8M	42.9M	36.6M

References

1. Lex, A., Gehlenborg, N., Strobelt, H., Vuillemot, R. & Pfister, H. UpSet: Visualization of Intersecting Sets. *IEEE Trans. Vis. Comput. Graph.* **20,** 1983–1992 (2014).
2. Sexton, T. *et al.* Three-dimensional folding and functional organization principles of the Drosophila genome. *Cell* **148,** 458–472 (2012).
3. Cubeñas-Potts, C. *et al.* Different enhancer classes in Drosophila bind distinct architectural proteins and mediate unique chromatin interactions and 3D architecture. *Nucleic Acids Res.* **45,** 1714–1730 (2017).
4. Wood, A. M. *et al.* Regulation of Chromatin Organization and Inducible Gene Expression by a Drosophila Insulator. *Mol. Cell* **44,** 29–38 (2011).
5. Li, L. *et al.* Widespread rearrangement of 3D chromatin organization underlies polycomb-mediated stress-induced silencing. *Mol. Cell* **58,** 216–231 (2015).
6. Ramírez, F. *et al.* deepTools2: a next generation web server for deep-sequencing data analysis. *Nucleic Acids Res.* **44,** W160–5 (2016).
7. Hug, C. B., Grimaldi, A. G., Kruse, K. & Vaquerizas, J. M. Chromatin Architecture Emerges during Zygotic Genome Activation Independent of Transcription. *Cell* **169,** 216–228.e19 (2017).
8. Iwasaki, O. *et al.* Interaction between TBP and Condensin Drives the Organization and Faithful Segregation of Mitotic Chromosomes. *Mol. Cell* **59,** 755–767 (2015).
9. Sanborn, A. L. *et al.* Chromatin extrusion explains key features of loop and domain formation in wild-type and engineered genomes. *Proc. Natl. Acad. Sci. U. S. A.* **112,**

E6456–65 (2015).

10. Goloborodko, A., Imakaev, M. V., Marko, J. F. & Mirny, L. Compaction and segregation of sister chromatids via active loop extrusion. *Elife* **5,** (2016).

11. Cook, P. R. A model for all genomes: the role of transcription factories. *J. Mol. Biol.* **395,** 1–10 (2010).

12. Sutherland, H. & Bickmore, W. A. Transcription factories: gene expression in unions? *Nat. Rev. Genet.* **10,** 457–466 (2009).

13. Stevens, T. J. *et al.* 3D structures of individual mammalian genomes studied by single-cell Hi-C. *Nature* **544,** 59–64 (2017).

14. Bailey, T. L. & Elkan, C. Fitting a mixture model by expectation maximization to discover motifs in biopolymers. *Proc. Int. Conf. Intell. Syst. Mol. Biol.* **2,** 28–36 (1994).

15. Bailey, T. L. DREME: motif discovery in transcription factor ChIP-seq data. *Bioinformatics* **27,** 1653–1659 (2011).

16. Li, L. *et al.* Widespread Rearrangement of 3D Chromatin Organization Underlies Polycomb-Mediated Stress-Induced Silencing. *Mol. Cell* **58,** 216–231 (2015).

17. Hou, C., Li, L., Qin, Z. S. & Corces, V. G. Gene density, transcription, and insulators contribute to the partition of the Drosophila genome into physical domains. *Mol. Cell* **48,** 471–484 (2012).

18. Ramírez, F. *et al.* High-Affinity Sites Form an Interaction Network to Facilitate Spreading of the MSL Complex across the X Chromosome in Drosophila. *Mol. Cell* **60,** 146–162 (2015).

19. Eagen, K. P., Hartl, T. A. & Kornberg, R. D. Stable Chromosome Condensation Revealed by Chromosome Conformation Capture. *Cell* **163,** 934–946 (2015).

20. Van Bortle, K. *et al.* Insulator function and topological domain border strength scale with architectural protein occupancy. *Genome Biol.* **15,** R82 (2014).

21. Zolotarev, N. *et al.* Architectural proteins Pita, Zw5,and ZIPIC contain homodimerization domain and support specific long-range interactions in Drosophila. *Nucleic Acids Res.* **44,** 7228–7241 (2016).

22. Li, J. & Gilmour, D. S. Distinct mechanisms of transcriptional pausing orchestrated by GAGA factor and M1BP, a novel transcription factor. *EMBO J.* **32,** 1829–1841 (2013).

23. Arnold, C. D. *et al.* Genome-wide quantitative enhancer activity maps identified by STARR-seq. *Science* **339,** 1074–1077 (2013).
