# Supplementary material for: High-resolution TADs reveal DNA sequences underlying genome organization in flies
Source: Nat Commun. 2018 Jan 15;9:189. doi: 10.1038/s41467-017-02525-w (PMC5768762; doi:10.1038/s41467-017-02525-w)
Supplement: Supplementary file 3 — Description of Additional Supplementary Files [file 41467_2017_2525_MOESM3_ESM.pdf]

Description of Additional Supplementary Files

File Name: Supplementary Data 1
Description: High-resolution TADs. *Drosophila melanogaster* (dm3) TADs classified as Active, Inactive, PcG or HP1 in bed format.
